# Bacterial colonisation during regular daily use of a power-driven water flosser and risk for cross-contamination. Can it be prevented?

**DOI:** 10.1007/s00784-021-04167-1

**Published:** 2021-09-18

**Authors:** Kristina Bertl, Chiarra Geissberger, David Zinndorf, Pia Edlund Johansson, Hatem Al-Shammari, Sigrun Eick, Andreas Stavropoulos

**Affiliations:** 1 Department of Periodontology, Faculty of Odontology, University of Malmö, Malmö, Sweden; 2 Division of Oral Surgery, University Clinic of Dentistry, Medical University of Vienna, Vienna, Austria; 3 Department of Periodontology, School of Dental Medicine, University of Bern, Bern, Switzerland; 4 Division of Regenerative Dental Medicine and Periodontology, University Clinics of Dental Medicine (CUMD), University of Geneva, Geneva, Switzerland; 5 Division of Conservative Dentistry and Periodontology, University Clinic of Dentistry, Medical University of Vienna, Vienna, Austria

**Keywords:** AirFloss, Bacterial colonisation, Cross-contamination, Disinfection, Interdental cleaning device, *Streptococcus mutans*

## Abstract

**Objective:**

To assess whether bacterial colonisation in a power-driven water flosser can be prevented.

**Materials and methods:**

Twenty-four patients undergoing supportive periodontal treatment used 2 power-driven water flossers [Sonicare AirFloss (SAF), AirFloss Ultra (SAFU)] for 12 weeks each as follows: (a) with bottled water (BW); (b) with BW and cleaning the device extra-orally twice per week with chlorhexidine gluconate or (c) essential-oil-based (EO) mouth-rinse; (d) with EO only. Water-jet samples were taken after 6 and 12 weeks with the used nozzle and after exchanging to a brand-new nozzle. After 12 weeks, all devices underwent an intensive cleaning procedure. Samples were analysed by PCR-based method for cariogenic and periodontal pathogens and culture for staphylococci, aerobe gram-negative bacteria, and *Candida* sp.

**Results:**

Contamination of SAF/SAFU with *Streptococcus mutans* was found in > 95% of the samples; periodontal pathogens and aerobe gram-negative bacteria were detected in 19–56% of the samples, while *Staphylococcus aureus* and *Candida* sp. were identified only in few samples. Contamination rate was basically unaffected by time-point, device, or way of use. Further, exchanging the nozzle did not prevent transmission of a contaminated water-jet, but the intensive cleaning reduced most of the pathogens significantly, except of *S. mutans*.

**Conclusion:**

Neither a specific way of use nor exchanging the nozzle prevented bacterial colonisation and transmission of biofilm components via the water-jet of SAF/SAFU.

**Clinical relevance:**

Bacterial colonisation in a power-driven water flosser seems impossible to prevent; to restrict the risk of cross-contamination within a household, one device per person should be recommended.

**Supplementary Information:**

The online version contains supplementary material available at 10.1007/s00784-021-04167-1.

## Introduction

Regular mechanical cleaning of the teeth including the daily use of a toothbrush together with an interdental cleaning aid is essential to minimise the risk of oral disease [1–5]. However, in order to achieve long-term success, patients have to comply daily with the chosen/recommended method; e.g. previous studies have shown, that power-driven interdental cleaning devices might be a preferred alternative for many patients [6–8]. Among these power-driven interdental cleaning devices, water flossers such as the Sonicare AirFloss or AirFloss Ultra (SAF/SAFU; Royal Philips N.V., Amsterdam, the Netherlands) have become popular. The SAF/SAFU emits a microburst of high velocity air and liquid micro-droplets, which is causing a sufficient shear stress on the interproximal tooth surface aiming to detach any biofilm accumulation [9, 10]. However, in terms of clinical parameters, the efficacy of SAF/SAFU remains still unclear [6, 7, 11, 12], but it appears indeed that it can achieve a higher acceptance among patients [6, 7].

A recent proof-of-principle study [13] reported that daily use of SAF for 3 weeks resulted in bacterial colonisation in the nozzle and/or device with both aerobic and anaerobic — not only oral — species, that are transmitted via the water-jet. Considering that bacterial colonisation has been previously reported for other oral hygiene devices (e.g., toothbrushes) [14–17], the finding that SAF is colonised by bacteria may come as no surprise, since the tip of the nozzle of the SAF comes in contact with the oral environment. Additionally, to transfer the water from the container to the nozzle and tip, the SAF contains an aqueous pipework; biofilm formation in aqueous pipework in general is a common phenomenon [18, 19]. However, such transmission of contaminated water-jet into the mouth may be a concern: (a) as potential source for re-infection during periodontal treatment similar to what has been discussed for microbial niches in the oral cavity other than periodontal pockets (e.g. tongue, tonsils) [20, 21], and (b) as potential source for cross-contamination among users similar to what was shown regarding cariogenic and periodontal bacteria from the mother to the child [22–24]. It is suggested that one device can be used by more than one person, with exchanging the nozzle as the only measure (i.e. one nozzle per person). If the source of the contaminated water-jet is biofilm accumulation only inside the nozzle, exchanging the nozzle would be an adequate protection measure in regard with the above concerns. However, if the biofilm accumulation is localized inside the device itself, exchanging the nozzle would not be a sufficient preventive measure. Since in the above-mentioned study [13] only water-jet samples transmitted via the used nozzle were analysed, no assumptions regarding the localization of the biofilm could be made.

The aim of the present study was to assess whether (a) biofilm in SAF/SAFU is localized in the nozzle and/or the device itself; (b) exchanging the nozzle is an adequate measure to prevent cross-contamination; (c) using SAF/SAFU exclusively with an essential-oil-based mouth-rinse (EO), and/or (d) rinsing the device regularly with a cleansing solution may inhibit/limit bacterial colonisation and/or effectively eliminate/limit the delivery of a contaminated water-jet.

## Material and methods

### Patient population

The present prospective cohort study was approved by the local Ethics Committee (Lund, Sweden; DNR 2014/388 & 2015/727), and was conducted in accordance with the Helsinki Declaration of 1975, as revised in 2013; reporting complies with the STROBE guidelines (Appendix 1). Twenty-four periodontitis patients, at the Dept. of Periodontology, Malmö University, Malmö, Sweden, fulfilling the following eligibility criteria and providing a signed informed consent were included: (a) had undergone non-surgical and/or surgical periodontal therapy, (b) were at timepoint of study recruitment already scheduled to supportive periodontal treatment (i.e. every 3–4 months); (c) had a minimum of 20 teeth; (d) provided ≥ 1 interproximal space between premolars or molars per quadrant; (e) had ≥ 5 residual interproximal periodontal pockets (i.e. ≥ 4 mm with bleeding on probing) within the whole dentition; (f) had no antibiotic intake in the last 3 months; (g) had no medication intake related to gingival hyperplasia; and (h) were not pregnant.

### Study outline

**Fig. 1:**
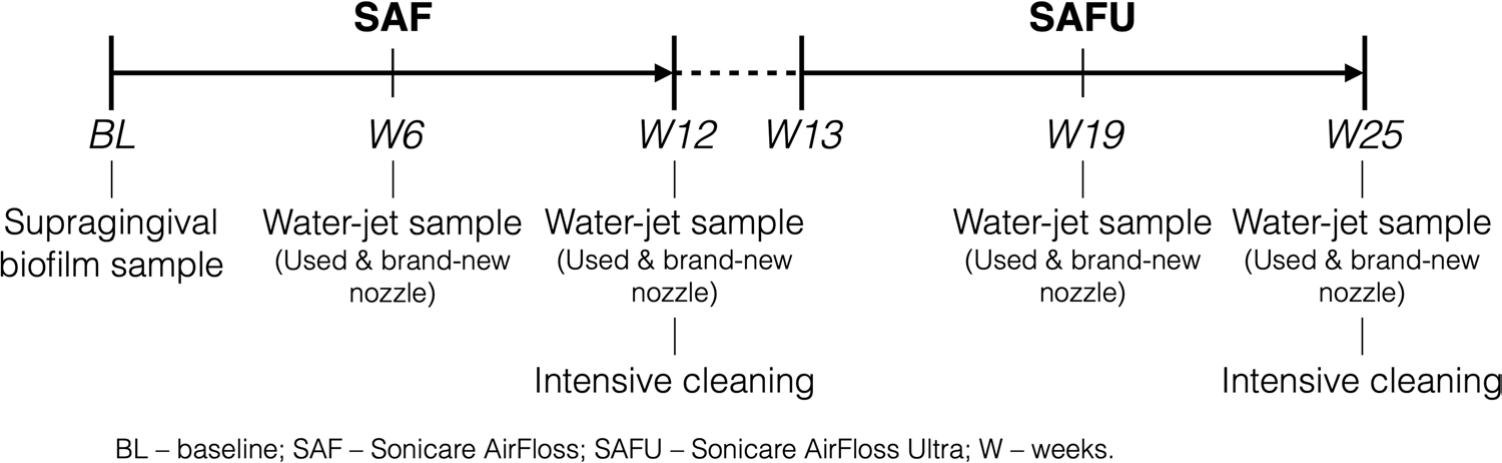
Study outline

The study included 2 consecutive experimental periods of 12 weeks, with a 1-week break in-between. The first period regarded the use of the first generation SAF (Royal Philips N.V., Amsterdam, the Netherlands) and the second the use of the second-generation SAFU (Royal Philips N.V., Amsterdam, the Netherlands); only brand-new devices were used. During each experimental period, the participants came back to the clinic once after 6 weeks for collecting water-jet samples of the devices and then after 12 weeks for returning the devices (Fig. 1). The devices were thereafter subjected to an intensive cleaning procedure (see “Intensive cleaning procedure”). Regular periodontal supportive treatment was delivered at baseline, after 12 weeks, and at the end of the study.

### Supragingival biofilm sample

At baseline, a supragingival biofilm sample was collected from one interproximal space between premolars or molars of each quadrant. The collection site was dried from saliva, and supragingival biofilm was collected with a sterile curette, transferred into a sterile tube (all 4 sites were pooled in the same tube), and stored at – 80 °C.

### Way of use of SAF/SAFU

At baseline, participants were instructed to use the SAF/SAFU once per day after tooth-brushing, by positioning the tip of the nozzle from the buccal aspect at each interproximal space and pressing the button once; thus, SAF delivered a single burst, while SAFU was pre-set to deliver a triple burst. Four different ways of use were assessed: (a) device used intra-orally only with bottled water (1 bottle/week; Evian®, Malmö, Sweden) (‘BW’); (b) device used intra-orally with bottled water (1 bottle/week) and twice per week performing a cleaning procedure extra-orally with chlorhexidine gluconate mouth-rinse (CHX; Flux Pro Chlorhexidin, 0.12%; Actavis Group PTC, Hafnarfjordur, Island) (‘BW + CHX’); (c) device used intra-orally with bottled water (1 bottle/week) and twice per week performing a cleaning procedure extra-orally with EO (‘BW + EO’; Listerine® Total Care, Johnson & Johnson Consumer Nordic); and (d) device used intra-orally only with EO (‘EO’). Bottled water was used to minimise the variation due to potential differences in the bacterial load of tap water among different city areas. Group allocation was based on 2 computer-generated randomization lists, one for the SAF and another for the SAFU. For the SAF, the 24 participants were randomly allocated to the following 3 groups: BW + CHX, BW + EO, and EO (i.e. 8 participants per group). Since it was clearly shown in the above-mentioned proof-of-principle study [13] that daily use with only bottled water results in biofilm formation in SAF, such a group (BW) was not included herein. For the SAFU, the participants were randomly allocated to all 4 treatment groups (i.e. 6 participants per group). In regard with the cleaning procedure (i.e. groups BW + CHX and BW + EO), the participants filled the container of the device twice per week with the mouth-rinse (i.e. CHX or EO) and used the device extra-orally until the container was empty, i.e. one full container of mouth-rinse was pressed through the waterpipe system and nozzle of the device. Further, the participants were instructed to rinse the nozzle only with the bottled water, to empty and swab the container with a clean tissue after each use, not to touch the nozzle with their fingers, not to drink from the bottled water, not to share the device among family members/partners, and not to exchange the nozzle (i.e. the same nozzle was used for 12 weeks). All participants were provided with a bag for transportation. During the study period, the participants were asked not to use any other interdental cleaning device or mouth-rinse.

### Water-jet sampling

Water-jet samples were collected after 6 and 12 weeks of daily use. The participants were instructed not to use the device 24 h before returning it. For water-jet sampling, the container was filled with bottled water and 2.5 ml water-jet samples were collected with the used or a brand-new nozzle into a sterile tube. Randomly, for half of the participants, water-jet sampling was done first with the used nozzle and then with the brand-new nozzle, while for the other half water-jet sampling was done in the reverse order. The sampling order was randomised to avoid any bias due to collecting the first 2.5 ml of the water-jet always with the same nozzle (i.e. either with the used or brand-new nozzle). Collection with the 2 types of nozzles facilitated identification of the source of contamination, i.e. whether the contamination is primarily in the nozzle or also in the device itself. Before exchanging the nozzle, the container was emptied, swabbed with a clean tissue, and filled again with fresh bottled water. All samples were stored at – 80 °C. After the first collection at 6 weeks, the used nozzle was mounted again, and the device was returned to the patient.

### Intensive cleaning procedure

After 12 weeks of daily use, an intensive cleaning procedure of the SAF/SAFU was performed as follows: (a) based on computer-generated randomization lists, separately for SAF and SAFU, the container was filled either with CHX or EO and the device was used extra-orally until the container was empty and this was repeated up to 40 times; (b) after the 10th, 20th, and 40th time, the container was filled once with bottled water and a 2.5-ml water-jet sample was collected, each time with a brand-new nozzle. All samples were stored at – 80 °C.

### Analysis of the supragingival biofilm and water-jet samples

Water samples were taken from the freezer and allowed to thaw up at 4 °C. The paper points samples were placed into 1 ml of 0.9% w/v NaCl. Then both, the tubes with water samples and paper points, were intensively vortexed. Thereafter, aliquots of the water and NaCl around the paper points were cultured on agar plates being selective for aerobe gram-negative rods (Mac Conkey agar, Oxoid, Basingstoke, GB), staphylococci (Mannitol Salt agar, Oxoid), and yeasts (CHROMID® Candida agar, bioMerieux). The respective microorganisms [i.e. aerobe gram-negative rods (e.g. enterobacteria, *Pseudomonas aeruginosa*), *Staphylococcus aureus*, and *Candida* sp.] were identified by colony, cell morphology, and biochemistry. Further, DNA was extracted from 1 ml of the water sample and the NaCl around the paper points by using Chelex technique [25]. Thereafter, real-time PCR was performed to count *Porphyromonas gingivalis*, *Tannerella forsythia*, *Treponema denticola*, *Fusobacterium nucleatum*, and *Streptococcus mutans* in the samples as described previously [26, 27].

### Statistical analysis

The samples were treated dichotomously (i.e. positive or negative for the specific pathogen) and as ordinal based on log_10_ values [i.e. (a) no detection, (b) < 3, (c) 3 to 6, and (d) ≥ 6]. Descriptive statistics for presenting frequencies and distributions, and chi-squared or Fisher’s exact test for testing of statistical significance of contamination rates among the various groups were used; Fisher’s exact test was used if one of the cells presented < 5 events. Except from when comparing the used vs. the brand-new nozzle, the device and nozzle were considered as one unit, i.e. irrespective whether a positive result was derived from the used or from the brand-new nozzle, the whole unit was judged as positive for this specific pathogen and time-point. Any comparisons of the actual log_10_ values were performed only for *S. mutans*. The normal distribution was confirmed by Q-Q-plots and comparisons of the mean were either performed by dependent (i.e. comparison between the contamination level at final evaluation and after the intensive cleaning procedure) or independent (i.e. comparison of the 2 mouth washes used for the intensive cleaning procedure) *t*-test. A statistical program (SPSS, Version 24.0, Chicago, IL, USA) was used and a *p*-value < 0.05 was considered statistically significant.

## Results

### Patient population

No remarkable events occurred during the first 12-week experimental period regarding the use of SAF. Out of the 24 patients (14 female, 10 male; age range 32 to 73 years, mean age 52.9 ± 11.2 years), 3 patients did not finish using the SAF exactly according to plan. Specifically, 2 of the patients reported that they did not use the device every single day, but they did not miss more than 7 days (i.e. the device was used for a total period of 11 weeks) and thus these data were included. The third patient had the nozzle of the SAF replaced after 6 weeks, due to a fracture of the nozzle resulting into water leakage during use; however, it was still possible to collect the water-jet sample. Two patients dropped out after using the SAF (1 patient did not return, and 1 patient started with orthodontic treatment). Additionally, several technical complications occurred during the second 12-week experimental period, regarding the use of SAFU, i.e. in several cases the device stopped working due to a defect battery either during the 12 weeks or during the intensive cleaning procedure. Specifically, 3 patients were excluded because the device broke already within the first 6 weeks, 3 patients contributed with only 6 weeks data because the device broke thereafter, while 2 patients finished the 12-week period using SAFU, but the device stopped working during the intensive cleaning procedure. This resulted in complete data for the SAFU for only 14 patients. One of those 14 patients reported not using the device every single day, but not missing more than 7 days in total; thus, these data were included in the analysis. Another patient reported taking antibiotics due to medical reasons during the 12-week period using the SAFU; however, the data of this patient were still included.

### Supragingival biofilm samples

**Fig. 2:**
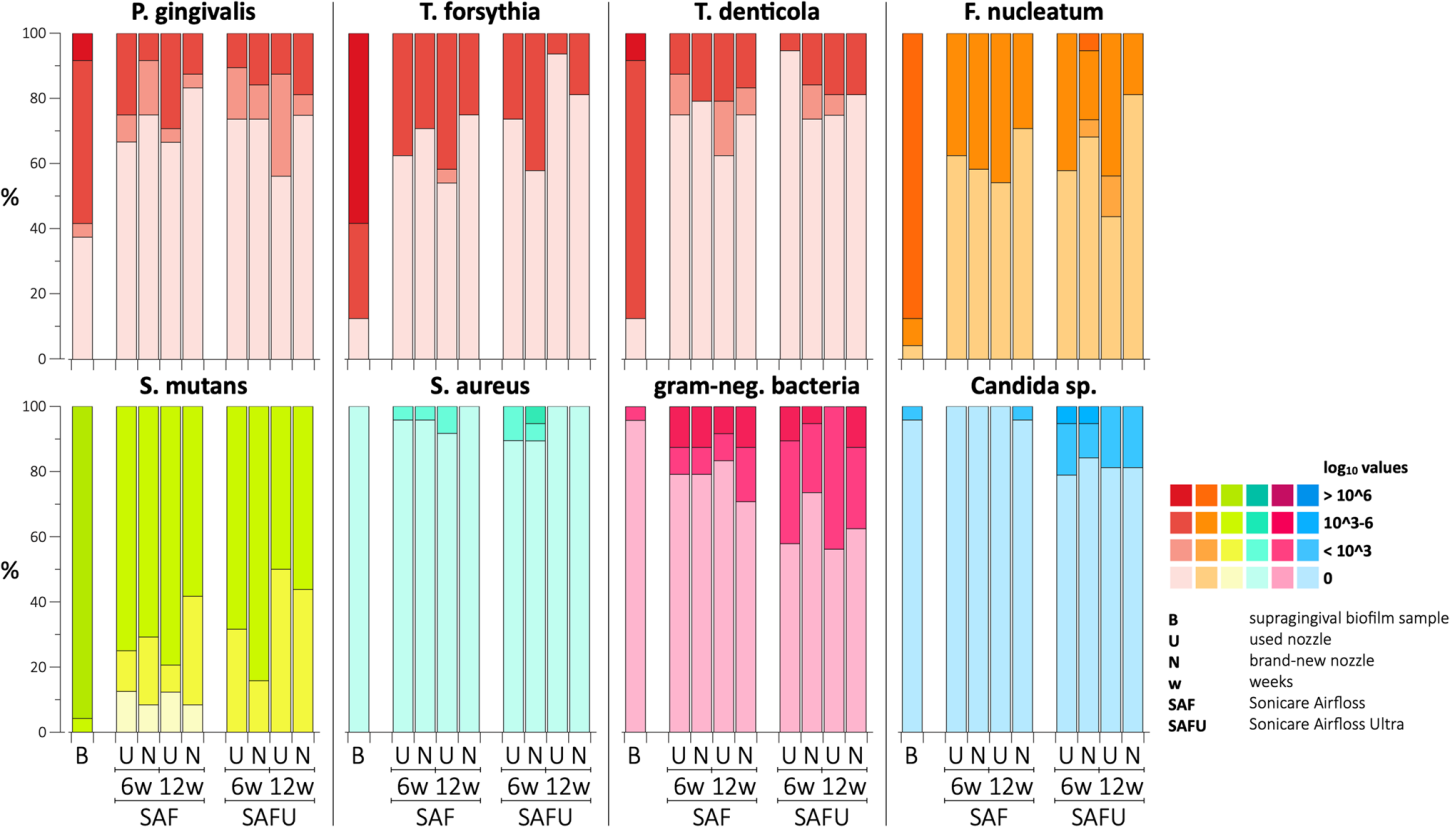
Contamination rate in supragingival biofilm and water-jet samples arranged per pathogen and their log_10_ values; latter were divided into 4 categories: (a) no detection, (b) < 3, (c) 3 to 6, and (d) ≥ 6

The supragingival biofilm samples from baseline (Fig. 2) were positive for all study participants for *S. mutans*; in one participant *F. nucleatum* was not detected, and in 3 participants, *T. denticola* and *T. forsythia* were not detected. Further, *P. gingivalis* was detected in > 60% of the samples, while aerobe gram-negative bacteria and *Candida* sp. were positive in only a single patient and *S. aureus* in none of the samples.

### Rates and type of bacterial contamination

Contamination rates and *p*-values are presented in Tables 1 and 2, and Appendix 2. Figure 2 shows contamination rates in supragingival biofilm and water-jet samples arranged per pathogen and their log_10_ values.

**Table 1:** Contamination rate of the water-jet samples with specific pathogens after 6 or 12 weeks using the SAF or SAFU, but independent of the nozzle (i.e. if one of the samples either from the used or brand-new nozzle was positive, the device was judged as positive for this specific pathogen and time-point)

**Device**	**Time-point**	**Pg**		**Tf**		**Td**		**Fn**		**Sm**		**Sa**		**Gram-neg**		**Candida**	
		***n***	***%***	***n***	***%***	***n***	***%***	***n***	***%***	***n***	***%***	***n***	***%***	***n***	***%***	***n***	***%***
**SAF**	*6 weeks*	9	37.5	11	45.8	7	29.2	12	50.0	23	95.8	1	4.2	5	20.8	0	0
	*12 weeks*	8	33.3	12	50.0	11	45.8	13	54.2	24	100	2	8.3	7	29.2	1	4.2
	*p-value (6 vs. 12 weeks)*	0.763		0.773		0.233		0.773		1.000		1.000		0.505		1.000	
**SAFU**	*6 weeks*	9	47.4	9	47.4	5	26.3	8	42.1	19	100	3	15.8	8	42.1	4	21.1
	*12 weeks*	7	43.8	3	18.8	4	25.0	9	56.3	16	100	0	0	9	56.3	4	25.0
	*p-value (6 vs. 12 weeks)*	0.830		0.152		1.000		0.404		–		0.234		0.404		1.000	
**SAF vs. SAFU**^***1***^	*p-value (at 6 weeks)*	*0.515*		*0.920*		*0.836*		*0.606*		*1.000*		*0.306*		*0.131*		***0.031***	
	*p-value (at 12 weeks)*	*0.505*		*0.056*		*0.318*		*0.897*		*–*		*0.508*		*0.087*		*0.138*	

*Fn*, Fusobacterium nucleatum; *gram-neg*., aerobe gram-negative bacteria; *Pg*, Porphyromonas gingivalis; *SAF*, Sonicare AirFloss; *SAFU*, Sonicare AirFloss Ultra; *Sa*, Staphylococcus aureus; *Sm*, Streptococcus mutans; *Td*, Treponema denticola; *Tf*, Tannerella forsythia

Bold values indicate statistical significance

^1^ Comparison of the contamination rate between SAF and SAFU device (chi squared test or Fisher’s exact test)

**Table 2:** Contamination rate among the 4 different ways of use

**Device**	**Time-point**	**Way of use (n)**	**Pg**		**Tf**		**Td**		**Fn**		**Sm**		**Sa**		**Gram-neg**		**Candida**	
			***n***	***%***	***n***	***%***	***n***	***%***	***n***	***%***	***n***	***%***	***n***	***%***	***n***	***%***	***n***	***%***
**SAF**	*6 weeks*	*BW* + *CHX (8)*	4	50.0	5	62.5	3	37.5	4	50.0	8	100	0	0	3	37.5	0	0
		*BW* + *EO (8)*	2	25.0	2	25.0	0	0	3	37.5	7	87.5	1	12.5	2	25.0	0	0
		*EO (8)*	3	37.5	4	50.0	4	50.0	5	62.5	8	100	0	0	0	0	0	0
		*p-value*^*1*^	0.587		0.309		0.073		0.607		0.352		0.352		0.171		–	
	*12 weeks*	*BW* + *CHX (8)*	2	25.0	5	62.5	5	62.5	5	62.5	8	100	1	12.5	3	37.5	0	0
		*BW* + *EO (8)*	3	37.5	4	50.0	3	37.5	3	37.5	8	100	1	12.5	4	50.0	1	12.5
		*EO (8)*	3	37.5	3	37.5	3	37.5	5	62.5	8	100	0	0	0	0	0	0
		*p-value*^*1*^	0.829		0.607		0.511		0.511		–		0.580		0.073		0.352	
**SAFU**	*6 weeks*	*BW (5)*	2	40.0	2	40.0	1	20.0	1	20.0	5	100	2	40.0	3	60.0	1	20.0
		*BW* + *CHX (6)*	4	66.7	4	66.7	0	0	4	66.7	6	100	0	0	3	50.0	2	33.3
		*BW* + *EO (6)*	3	50.0	3	50.0	3	50.0	3	50.0	6	100	1	16.7	2	33.3	1	16.7
		*EO (2)*	0	0	0	0	1	50	0	0	2	100	0	0	0	0	0	0
		*p-value*^*1*^	0.420		0.420		0.207		0.251		–		0.295		0.484		0.765	
	*12 weeks*	*BW (4)*	2	50.0	0	0	2	50.0	2	50.0	4	100	0	0	2	50.0	0	0
		*BW* + *CHX (5)*	3	60.0	1	20.0	0	0	3	60.0	5	100	0	0	4	80.0	4	80.0
		*BW* + *EO (6)*	2	33.3	2	33.3	2	33.3	4	66.7	6	100	0	0	3	50.0	0	0
		*EO (1)*	0	0	0	0	0	0	0	0	1	100	0	0	0	0	0	0
		*p-value*^*1*^	0.650		0.573		0.314		0.650		–		–		0.459		**0.008**	

*Fn*, Fusobacterium nucleatum; *gram-neg*., aerobe gram-negative bacteria; *Pg*, Porphyromonas gingivalis; *SAF*, Sonicare AirFloss; *SAFU*, Sonicare AirFloss Ultra; *Sa*, Staphylococcus aureus; *Sm*, Streptococcus mutans; *Td*, Treponema denticola; *Tf*, Tannerella forsythia

Treatment groups: ‘BW’ — device used only with bottled water; ‘BW + CHX’ — device used with bottled water and twice per week performing a cleaning procedure with chlorhexidine gluconate mouth-rinse; ‘BW + EO’ — device used with bottled water and twice per week performing a cleaning procedure with essential-oil-based mouth-rinse; ‘EO’ — device used only with essential-oil-based mouth-rinse

Bold values indicate statistical significance

^1^ Chi squared test among different treatment regimens

#### Water-jet samples — 6 vs. 12 weeks

All water-jet samples were contaminated with at least one of the tested pathogens already after 6 weeks. After 12 weeks and independent whether SAF or SAFU was used, *S. mutans* was detected in 100% of the cases, *P. gingivalis*, *T. forsythia*, *T. denticola*, *F. nucleatum*, and aerobe gram-negative bacteria were detected in 18.8% to 56.3% of the cases, while *Candida* sp. and *S. aureus* were detected in ≤ 25% of the cases. No statistically significant differences were found regarding the contamination rate of water-jet samples between 6 and 12 weeks (SAF *p* ≥ 0.233, SAFU *p* ≥ 0.152; Table 1). Further, no statistically significant differences were found at 6 or 12 weeks regarding the rate and/or the type of bacterial contamination between SAF or SAFU (*p* ≥ 0.056), except for *Candida* sp. after 6 weeks (*p* = 0.031), which was more often detected in SAFU (Table 1). In general, the number of pathogens was lower in the water-jet samples compared to the supragingival biofilm samples for *P. gingivalis*, *T. forsythia*, *T. denticola*, *F. nucleatum*, and *S. mutans*, but the water-jet samples presented still in most of the cases log_10_ values between 3 and 6 (Fig. 2).

#### Water-jet samples — way of use of SAF/SAFU

None of the ways of use of SAF showed any tendency for lower rates of contamination, while the use of SAFU with EO showed no contamination regarding several of the tested bacteria. However, due to the technical problems mentioned above, there were only 2 and 1 devices available for analysis after 6 and 12 weeks, respectively, which had been used with only EO. Thus, except of a significantly higher contamination rate with *Candida* sp. after 12 weeks in the BW + CHX group of SAFU, the way of use of SAF or SAFU did not appear having any significant impact on the contamination rate of the devices (*p* ≥ 0.073; Table 2).

#### Water-jet samples — used vs. brand-new nozzle

No significant differences in rate and type of bacterial contamination were seen between water-jet samples delivered from used and brand-new nozzles, irrespective of the time-point (i.e. 6 or 12 weeks) and device (i.e. SAF or SAFU) (*p* ≥ 0.131; Appendix 2).

### Intensive cleaning procedure

**Fig. 3:**
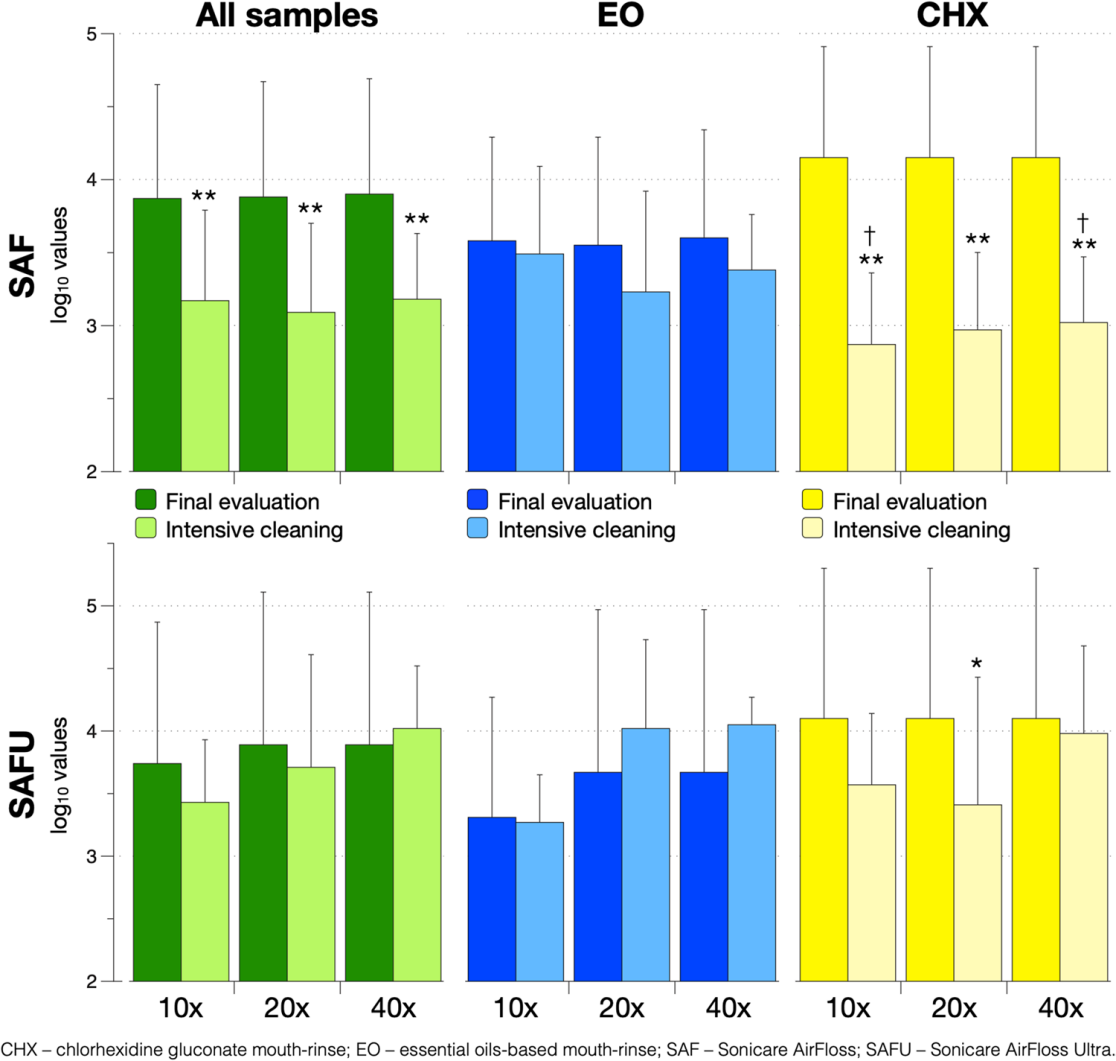
Effect of the intensive cleaning procedure (i.e. 10, 20 and 40 cleaning terms) on *Streptococcus mutans* (log_10_ values; mean ± S.D.). * (*p* < 0.05) / ** (*p* < 0.01) significantly lower values compared to the samples taken at final evaluation; † (*p* < 0.05) significantly lower values than the corresponding values with EO as cleaning agent

In general, contamination rate for the various tested pathogens — except for *S. mutans* — was reduced by 60 to 100% in the SAF and by 0 to 100% in the SAFU with the intensive cleaning procedure (Table 3). CHX and EO were equally effective (*p* ≥ 0.462; Appendix 3) in reducing statistically significant the contamination rate in both — SAF and SAFU — already after the first 10 cleaning times (Table 3); extending the cleaning procedure to 20- or 40-times did not show any significant additional benefit (*p* ≥ 0.348). *S. mutans* was almost unaffected by the intensive cleaning procedure and contamination rate stayed above 90% even after 40 cleaning times (Table 3 and Appendix 3). However, in terms of the log_10_ values of *S. mutans*, a reduction after the intensive cleaning procedure was noted, which reached statistically significance for the SAF device; this reduction was more pronounced with using CHX compared to EO (Fig. 3).

**Table 3:** Contamination rate after the intensive cleaning procedure based on 24 SAF and 14 SAFU devices, but independent of the cleaning agent used (i.e. the data of both cleaning agents were combined)

**Device** **(n)**	**No. of cleaning terms**		**Pg**		**Tf**		**Td**		**Fn**		**Sm**		**Sa**		**Gram-neg**		**Candida**	
			***n***	***%***	***n***	***%***	***n***	***%***	***n***	***%***	***n***	***%***	***n***	***%***	***n***	***%***	***n***	***%***
**SAF** **(24)**	*Contamination rate at FE*^*1*^		8	33.3	12	50.0	11	45.8	13	54.2	24	100	2	8.3	7	29.2	1	4.2
	*10x*	*% reduction from FE*^*3*^	− 62.5%		** − 75%**		− 63.6%		** − 84.6%**		− 4.2%		− 100%		** − 100%**		− 100%	
		*p-value*^*2*^	0.168		**0.011**		0.060		**0.001**		1.000		0.489		**0.009**		1.000	
	*20x*	*% reduction from FE*^*3*^	− 75%		** − 91.7%**		** − 81.8%**		** − 84.6%**		− 8.3%		− 100%		** − 100%**		− 100%	
		*p-value*^*2*^	0.072		**0.001**		**0.008**		**0.001**		0.489		0.489		**0.009**		1.000	
	*40x*	*% reduction from FE*^*3*^	** − 87.5%**		** − 75%**		** − 90.9%**		** − 92.3%**		− 8.3%		− 100%		** − 100%**		− 100%	
		*p-value*^*2*^	**0.023**		**0.011**		**0.002**		** < 0.001**		0.489		0.489		**0.009**		1.000	
**SAFU** **(14)**	*Contamination rate at FE*^*1*^		6	42.9	3	21.4	3	21.4	8	57.1	14	100	0	0	8	57.1	4	28.6
	*10x*	*% reduction from FE *^*3*^	− 83.3%		− 33.3%		− 66.7%		** − 75%**		− 7.1%		−		** − 100%**		− 100%	
		*p-value*^*2*^	0.077		1.000		0.596		**0.046**		1.000		–		**0.002**		0.098	
	*20x*	*% reduction from FE *^*3*^	** − 100%**		0%		− 100%		− 62.5%		0%		–		** − 87.5%**		− 100%	
		*p-value*^*2*^	**0.016**		1.000		0.222		0.120		–		–		**0.013**		0.098	
	*40x*	*% reduction from FE *^*3*^	− 50%		− 33.3%		− 33.3%		− 62.5%		0%		–		** − 100%**		− 100%	
		*p-value*^*2*^	0.420		1.000		1.000		0.120		–		–		**0.002**		0.098	

*CHX*, chlorhexidine gluconate mouth wash; *EO*, essential oil-based mouth wash; *FE*, final evaluation after 12 weeks, *Fn*, Fusobacterium nucleatum; *gram-neg*., aerobe gram-negative bacteria; *Pg*, Porphyromonas gingivalis; *SAF*, Sonicare AirFloss; *SAFU*, Sonicare AirFloss Ultra; *Sa*, Staphylococcus aureus; *Sm*, Streptococcus mutans; *Td*, Treponema denticola; *Tf*, Tannerella forsythia

Bold values indicate statistical significance

^1^ Independent of the nozzle

^2^ Comparison of the contamination rate at FE and after 10-, 20-, or 40-times of intensive cleaning independent of the cleaning agent

^3^ Data of both cleaning agents were combined

## Discussion

The results of the present study have shown that both first and second generation Airfloss devices are permanently colonised by oral bacteria — especially with *S. mutans* — already after 6 weeks of regular use, irrespective of using the device with water or exclusively with a mouth-rinse and irrespective of any special cleaning procedure. Further, these bacteria are transmitted via the water-jet and exchanging the used nozzle to a brand-new one did not prevent transmission of bacteria from the device via the water-jet, i.e. there is always a risk for cross-contamination if using the same device.

Bacterial colonisation and transmission via the water-jet was tested in SAF and SAFU after regular daily use for 12 weeks. Similar to the pilot trial with only the SAF [13], the colonisation appeared early, i.e. already after 6 weeks both type of devices herein were colonised with at least one of the tested pathogens and the contamination rate hardly changed over time. The highest contamination rate (i.e. in > 95% of the cases) was shown for a caries-associated pathogen (i.e. *S. mutans*), which is in general well-known for its ability to form highly resistant biofilms [28]. Further, periodontal pathogens, such as *P. gingivalis*, *T. forsythia*, *T. denticola*, and *F. nucleatum*, were detected in up to 60% of the cases. *S. aureus*, which frequently colonises the skin and/or the upper respiratory tract, and *Candida* sp. were only seldomly detected. Hence, although various sources for bacterial contamination of the SAF/SAFU are plausible (i.e. from the oral cavity, from the skin, etc.), it appears that primarily *S. mutans* and periodontal pathogens account for the colonisation, while *Candida* sp., and *S. aureus* take only a minor proportion.

In the pilot trial [13], it was shown that already after 3 weeks of regular daily use of SAF only with bottled water, bacterial colonisation occurred. Thus, it was of interest to assess, whether a specific way of use can prevent bacterial colonisation in the device. Several options have been tested herein: (a) single vs. triple burst of the water-jet, (b) application of any additional cleaning procedures (i.e. in a simple way on a weekly base or a more intensive cleaning procedure after established bacterial colonisation), and (c) using the device with a mouth-rinse instead of water. However, none of the tested methods showed a significant potential to inhibit/limit bacterial colonisation in the device and/or to eliminate/limit the delivery of a contaminated water-jet. Specifically, the second generation of this specific power-driven water flosser (i.e. SAFU) offers the possibility to work with a triple instead of single water-jet burst. One could hypothesize that this causes higher shear forces within the device and nozzle, which in turn potentially does not allow or reduces initial biofilm build-up. Herein, the SAFU was pre-set to a triple water-jet burst, but the comparison of the SAF and SAFU hardly presented any statistically significant differences. In fact, the only statistical significance detected favoured the first generation of the device (i.e. the SAF) due to a lower contamination rate with *Candida* sp. after 6 weeks; however, this finding should not be overrated since the contamination rate with *Candida* sp. was in general low. More interestingly, none of the different ways of use prevented bacterial colonisation, and only an intensive cleaning procedure showed some positive effects. Specifically, all tested pathogens except of *S. mutans* were reduced already after the first 10 cleaning terms at a statistically significant level. However, neither an extension of the cleaning procedure (i.e. up to 20 or 40 cleaning terms) had any additional benefit nor the type of the mouth-rinse (i.e. CHX or EO) made a difference. The contamination rate with *S. mutans* was almost unaffected by the intensive cleaning procedure and stayed above 90% even after 40 cleaning terms; only a slight, but for the SAF statistically significant reduction in the log_10_ values of *S. mutans* was determined, which appeared to be mainly due to the effect of CHX. Altogether, although the contamination rate of most pathogens tested herein could be reduced with an intensive cleaning procedure, the main contaminating pathogen (i.e. *S. mutans*) was largely unaffected. *S. mutans* synthesizes glucans which enables to produce a biofilm matrix being highly resistant against antimicrobials [28].

**Fig. 4:**
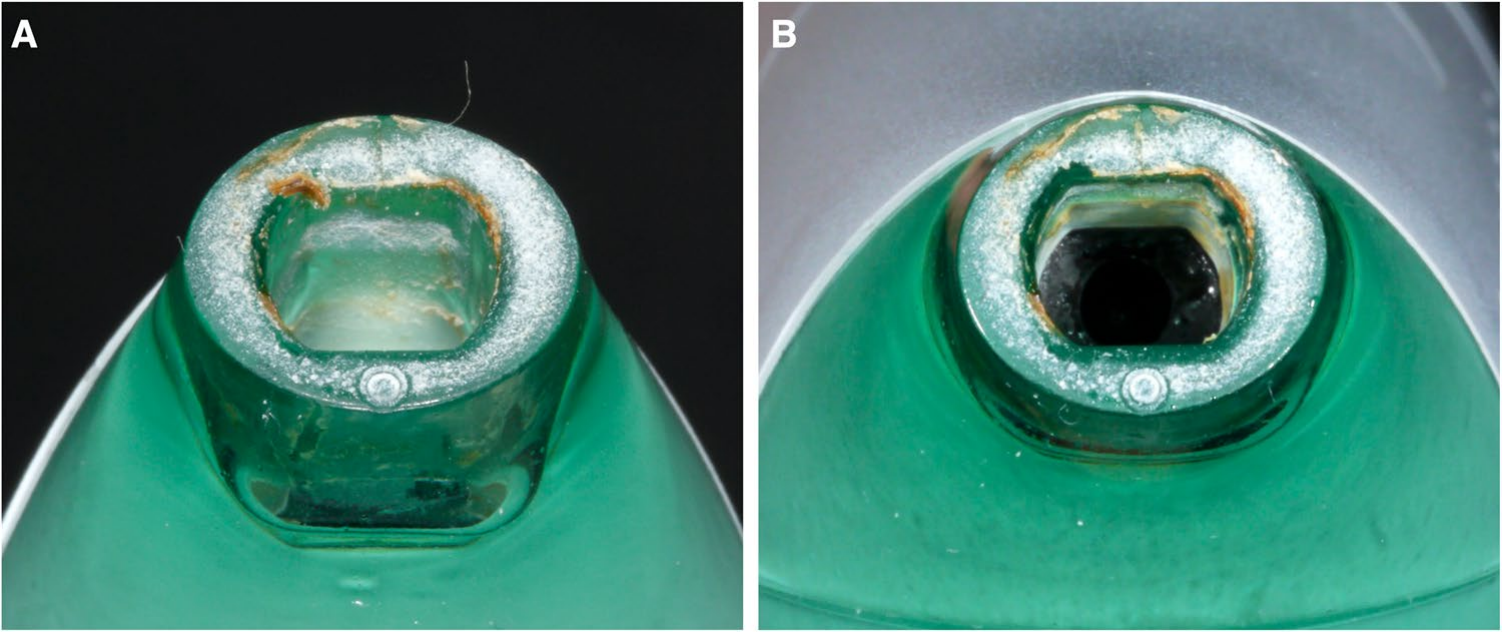
Close-up photographs of the nozzle connection of 2 different SAF 12 weeks after regular daily use showing biofilm formation inside the device

Colonisation with oral bacteria is reported also for other oral hygiene products, such as toothbrushes [14–17]. However, while toothbrushes are regularly not shared among different persons, SAF and SAFU are supposed to be used by more than one family/house-hold member with the only precaution to exchange the nozzle. However, exchanging the used nozzle to a brand-new one did not prevent transmission of biofilm components via the water-jet, i.e. irrespective of the time-point and device, the water-jet samples derived from the brand-new nozzle presented a comparable contamination rate as the samples from the used nozzle. This implies, that the bacterial colonisation of the device does not take place only in the nozzle, but also in the device itself (Fig. 4). In this context, the question remains, whether once or twice daily transmission of biofilm components can actually cause a permanent change of the oral microbiota of another person. It was shown previously, that even intimate kissing with an estimated transfer of 80 million bacteria within 10 s, “requires” a relatively high daily frequency to cause a certain degree of shared salivary microbiota [29]. Nevertheless, taking the present results into account, the recommendation to use one nozzle per person within one household should be reconsidered. Particularly, sharing the device among parents and children should be avoided to minimise the risk of vertical transmission of *S. mutans* [22, 30], especially considering the high contamination rate observed herein, but also of periodontal pathogens [23, 24, 31]. Further, one should also keep in mind, that exchanging the nozzle does not eliminate the risk of re-infection for example in patients continuing to use the same device after initiating periodontal treatment.

The present study faced some technical issues with the second generation of devices, which resulted in a limited number of participants per group in the second run. However, based on the lack of significant differences between the SAF and SAFU in general as well as based on the 100% contamination rate with *S. mutans*, it appears correct to conclude that none of the ways of use could prevent colonisation, despite the relatively limited sample size and the high standard deviation among the log_10_ values. Future studies might address, whether a daily (intensive) cleaning procedure after every use could prevent biofilm build-up. However, it is questionable whether such an approach would meet a high compliance rate among the patients on the long-term.

In conclusion, bacterial colonisation in a power-driven water flosser and transmission of contaminated water-jet — especially in terms of the main caries-associated pathogen *S. mutans* — seems impossible to prevent. Neither using the device exclusively with a mouth-rinse nor any cleaning procedures prevented bacterial colonisation within the device and failed to disinfect the device — especially regarding *S. mutans*. Further, exchanging the used nozzle to a brand-new one did not prevent the risk of cross-contamination, i.e. bacteria from the device were also transmitted via the water-jet of a brand-new nozzle. Hence, to restrict the risk of cross-contamination within a household, one device per person should be recommended.

## Supplementary Information

Below is the link to the electronic supplementary material.

Supplementary file1 (DOC 85 KB)

Supplementary file2 (DOCX 19 KB)

Supplementary file3 (DOCX 22 KB)
