# Supplementary material for: Bacterial colonisation during regular daily use of a power-driven water flosser and risk for cross-contamination. Can it be prevented?
Source: Clin Oral Investig. 2021 Sep 18;26(2):1903–13. doi: 10.1007/s00784-021-04167-1 (PMC8816322; doi:10.1007/s00784-021-04167-1)
Supplement: Supplementary file 2 — Supplementary file2 (DOCX 19 KB) [file 784_2021_4167_MOESM2_ESM.docx]

**Appendix 2.** Assessment of the risk for cross-contamination (i.e., comparison of the contamination rate of water-jet samples derived either from a used or brand-new nozzle).

| **Device** | **Time-point** | **Nozzle** | **Pg** | | **Tf** | | **Td** | | **Fn** | | **Sm** | | **Sa** | | **Gram-neg.** | | **Candida** | |
| --- | --- | --- | --- | --- | --- | --- | --- | --- | --- | --- | --- | --- | --- | --- | --- | --- | --- | --- |
|  |  |  | ***n*** | ***%*** | ***n*** | ***%*** | ***n*** | ***%*** | ***n*** | ***%*** | ***n*** | ***%*** | ***n*** | ***%*** | ***n*** | ***%*** | ***n*** | ***%*** |
| **SAF** | *6 weeks* | *Used* | 8 | 33.3 | 9 | 37.5 | 6 | 25.0 | 9 | 37.5 | 21 | 87.5 | 1 | 4.2 | 5 | 20.8 | 0 | 0 |
|  |  | *Brand-new* | 6 | 25.0 | 7 | 29.2 | 5 | 20.8 | 10 | 41.7 | 22 | 91.7 | 1 | 4.2 | 5 | 20.8 | 0 | 0 |
|  |  | *p-value^1^* | *0.525* | | *0.540* | | *0.731* | | *0.768* | | *1.000* | | *1.000* | | *1.000* | | *-* | |
|  | *12 weeks* | *Used* | 8 | 33.3 | 11 | 45.8 | 9 | 37.5 | 11 | 45.8 | 21 | 87.5 | 2 | 8.3 | 4 | 16.7 | 0 | 0 |
|  |  | *Brand-new* | 4 | 16.7 | 6 | 25.0 | 6 | 25.0 | 7 | 29.2 | 22 | 91.7 | 0 | 0 | 7 | 29.2 | 1 | 4.2 |
|  |  | *p-value^1^* | *0.318* | | *0.131* | | *0.350* | | *0.233* | | *1.000* | | *0.489* | | *0.494* | | *1.000* | |
| **SAFU** | *6 weeks* | *Used* | 5 | 26.3 | 5 | 26.3 | 1 | 5.3 | 8 | 42.1 | 19 | 100 | 2 | 10.5 | 8 | 42.1 | 4 | 21.1 |
|  |  | *Brand-new* | 5 | 26.3 | 8 | 42.1 | 5 | 26.3 | 6 | 31.6 | 19 | 100 | 2 | 10.5 | 5 | 26.3 | 3 | 15.8 |
|  |  | *p-value^1^* | *1.000* | | *0.305* | | *0.180* | | *0.501* | | *-* | | *1.000* | | *0.305* | | *1.000* | |
|  | *12 weeks* | *Used* | 7 | 43.8 | 1 | 6.3 | 2 | 25.0 | 9 | 56.3 | 16 | 100 | 0 | 0 | 7 | 43.8 | 3 | 18.8 |
|  |  | *Brand-new* | 4 | 25.0 | 3 | 18.8 | 3 | 18.8 | 3 | 18.8 | 16 | 100 | 0 | 0 | 6 | 37.5 | 3 | 18.8 |
|  |  | *p-value^1^* | *0.458* | | *0.600* | | *1.000* | | *0.066* | | *-* | | *-* | | *0.719* | | *1.000* | |

*Fn – Fusobacterium nucleatum; gram-neg. – aerobe gram-negative bacteria; Pg – Porphyromonas gingivalis; SAF – Sonicare AirFloss; SAFU – Sonicare AirFloss Ultra; Sa – Staphylococcus aureus; Sm – Streptococcus mutans; Td – Treponema denticola; Tf – Tannerella forsythia.*

*^1^ Comparison of the contamination rate between the used and the brand-new nozzle (Chi squared test or Fisher’s exact test).*
