# Supplementary material for: Bacterial colonisation during regular daily use of a power-driven water flosser and risk for cross-contamination. Can it be prevented?
Source: Clin Oral Investig. 2021 Sep 18;26(2):1903–13. doi: 10.1007/s00784-021-04167-1 (PMC8816322; doi:10.1007/s00784-021-04167-1)
Supplement: Supplementary file 3 — Supplementary file3 (DOCX 22 KB) [file 784_2021_4167_MOESM3_ESM.docx]

**Appendix 3.** Comparison of the contamination rate between the cleaning agents based on 24 SAF and 14 SAFU devices.

| **Device**  **(n)** | **No. of cleaning terms** | **Cleaning agent** | **Pg** | | **Tf** | | **Td** | | **Fn** | | **Sm** | | **Sa** | | **Gram-neg.** | | **Candida** | |
| --- | --- | --- | --- | --- | --- | --- | --- | --- | --- | --- | --- | --- | --- | --- | --- | --- | --- | --- |
|  |  |  | ***n*** | ***%*** | ***n*** | ***%*** | ***n*** | ***%*** | ***n*** | ***%*** | ***n*** | ***%*** | ***n*** | ***%*** | ***n*** | ***%*** | ***n*** | ***%*** |
| **SAF**  **(24)** | *Contamination rate at FE^1^* | | 8 | 33.3 | 12 | 50.0 | 11 | 45.8 | 13 | 54.2 | 24 | 100 | 2 | 8.3 | 7 | 29.2 | 1 | 4.2 |
|  | *10x* | *EO* | 2 | 16.7 | 2 | 16.7 | 2 | 16.7 | 2 | 16.7 | 11 | 91.7 | 0 | 0 | 0 | 0 | 0 | 0 |
|  |  | *CHX* | 1 | 8.3 | 1 | 8.3 | 2 | 16.7 | 0 | 0 | 12 | 100 | 0 | 0 | 0 | 0 | 0 | 0 |
|  |  | *p-value^2^* | 1.000 | | 1.000 | | 1.000 | | 0.478 | | 1.000 | | - | | - | | - | |
|  | *20x* | *EO* | 1 | 8.3 | 0 | 0 | 1 | 8.3 | 1 | 8.3 | 10 | 83.3 | 0 | 0 | 0 | 0 | 0 | 0 |
|  |  | *CHX* | 1 | 8.3 | 1 | 8.3 | 1 | 8.3 | 1 | 8.3 | 12 | 100 | 0 | 0 | 0 | 0 | 0 | 0 |
|  |  | *p-value^2^* | 1.000 | | 1.000 | | 1.000 | | 1.000 | | 0.478 | | - | | - | | - | |
|  | *40x* | *EO* | 0 | 0 | 1 | 8.3 | 1 | 8.3 | 1 | 8.3 | 10 | 83.3 | 0 | 0 | 0 | 0 | 0 | 0 |
|  |  | *CHX* | 1 | 8.3 | 2 | 16.7 | 0 | 0 | 0 | 0 | 12 | 100 | 0 | 0 | 0 | 0 | 0 | 0 |
|  |  | *p-value^2^* | 1.000 | | 1.000 | | 1.000 | | 1.000 | | 0.478 | | - | | - | | - | |
| **SAFU**  **(14)** | *Contamination rate at FE^1,3^* | | 6 | 42.9 | 3 | 21.4 | 3 | 21.4 | 8 | 57.1 | 14 | 100 | 0 | 0 | 8 | 57.1 | 4 | 28.6 |
|  | *10x* | *EO* | 1 | 14.3 | 1 | 14.3 | 1 | 14.3 | 1 | 14.3 | 6 | 85.7 | 0 | 0 | 0 | 0 | 0 | 0 |
|  |  | *CHX* | 0 | 0 | 1 | 14.3 | 0 | 0 | 1 | 14.3 | 7 | 100 | 0 | 0 | 0 | 0 | 0 | 0 |
|  |  | *p-value^2^* | 1.000 | | 1.000 | | 1.000 | | 1.000 | | 1.000 | | - | | - | | - | |
|  | *20x* | *EO* | 0 | 0 | 2 | 28.6 | 0 | 0 | 1 | 14.3 | 7 | 100 | 0 | 0 | 1 | 14.3 | 0 | 0 |
|  |  | *CHX* | 0 | 0 | 1 | 14.3 | 0 | 0 | 2 | 28.6 | 7 | 100 | 0 | 0 | 0 | 0 | 0 | 0 |
|  |  | *p-value^2^* | - | | 1.000 | | - | | 1.000 | | - | | - | | 1.000 | | - | |
|  | *40x* | *EO* | 1 | 14.3 | 2 | 28.6 | 1 | 14.3 | 2 | 28.6 | 7 | 100 | 0 | 0 | 0 | 0 | 0 | 0 |
|  |  | *CHX* | 2 | 28.6 | 0 | 0 | 1 | 14.3 | 1 | 14.3 | 7 | 100 | 0 | 0 | 0 | 0 | 0 | 0 |
|  |  | *p-value^2^* | 1.000 | | 0.462 | | 1.000 | | 1.000 | | - | | - | | - | | - | |

*CHX – chlorhexidine gluconate mouth wash; EO – essential oil-based mouth wash; FE – final evaluation after 12 weeks, Fn – Fusobacterium nucleatum; gram-neg. – aerobe gram-negative bacteria; Pg – Porphyromonas gingivalis; SAF – Sonicare AirFloss; SAFU – Sonicare AirFloss Ultra; Sa – Staphylococcus aureus; Sm – Streptococcus mutans; Td – Treponema denticola; Tf – Tannerella forsythia.*

*Bold values indicate statistical significance.*

*^1^ Independent of the nozzle.*

*^2^ Comparison of the contamination rate between the cleaning agents (Fisher’s exact test).*

*^3^ Based on the 14 devices for which an intensive cleaning procedure could be performed.*
